# A qualitative study to explore symptoms and impacts of pediatric and adolescent Crohn’s disease from patient and caregiver perspective

**DOI:** 10.1186/s41687-021-00321-1

**Published:** 2021-06-25

**Authors:** Louise Newton, Laure Delbecque, Ufuk Coşkun, Tara Symonds, Jennifer Clegg, Theresa Hunter

**Affiliations:** 1 Clinical Outcomes Solutions, Folkestone, UK; 2 Eli Lilly and Company, Brussels, Belgium; 3 Clinical Outcomes Solutions, Tucson, AZ USA; 4 Clinical Outcomes Solutions, 53 W Jackson Blvd, Ste 1150, Chicago, IL 60604 USA; 5 Eli Lilly and Company, Indianapolis, IN USA

**Keywords:** Crohn’s disease, Pediatric, Adolescent, Parent, Caregiver, Observer, Concept elicitation, Qualitative, Interview

## Abstract

**Background:**

Crohn’s disease (CD) is a chronic inflammatory condition of the gastrointestinal tract that affects people across the age spectrum but often starts in childhood or early adulthood. Despite this, almost all published research examining the symptomatic and health-related quality of life (HRQL) experiences of CD has been conducted in an adult population. Studies providing a comprehensive overview of the lived experience of pediatric and adolescent CD are virtually non-existent. The experiences of younger children aged 2–7 years are especially unknown.

**Results:**

A total of 49 participants (31 children and 18 parents) were interviewed. This included 11 dyads (i.e., parents and children from the same family). Analyses were conducted based on reporter-type (patient self-report vs parent observer-report) and age subgroups (ages 2–4 vs 5–7 vs 8–11 vs 12–17). Key symptoms were identified across the age subgroups and reporter types. Abdominal/stomach pain, passing gas/feeling gassy, diarrhea/liquid stools, fatigue/tiredness, bowel urgency, blood in stools, stomach cramping, constipation, and incomplete evacuation were discussed most frequently. The most common HRQL impacts included impact on physical activity, school, social life, and mood (i.e., feeling sad/low), and were mostly consistent between reporter type and across age spectrum. Concept agreement between parents and children in the dyad analysis was > 60% for most symptoms and impacts.

**Conclusions:**

Qualitative interviews revealed the substantial symptom and HRQL burden of pediatric CD from the child and parent perspectives and that disease experiences were largely consistent across the age range and based on both reporter perspectives. This is an important first step towards implementing a robust measurement strategy for the assessment of symptoms and HRQL impacts in pediatric CD.

**Supplementary Information:**

The online version contains supplementary material available at 10.1186/s41687-021-00321-1.

## Background

Crohn’s disease (CD) is an inflammatory bowel disease that causes chronic inflammation which can affect any part of the gastrointestinal (GI) tract. The disease course is characterized by periods of clinical remission and relapse and is usually progressive in nature [1]. Typical symptoms of CD include persistent diarrhea/liquid stools, abdominal pain, fatigue, rectal bleeding, appetite loss, weight loss, and perianal disease [2]. As a result of these symptoms, CD can have a significant impact on a person’s health-related quality of life (HRQL) including their sleep, daily activities, social and leisure activities, as well as emotional and psychological factors [3].

Crohn’s disease can affect a person of any age although diagnosis most often occurs in young adulthood [4]. Prevalence data indicate that in Western Europe and North America, the number of cases is between 100 to 300 per 100,000 people [5]. The highest annual incidences are 20.2/100,000 in North America and 12.7/100,000 in Europe [6], and for pediatric CD specifically, 13.9/100,000 in North America and 12.3/100,000 in Europe [5]. Moreover, studies have shown that patients diagnosed with CD in childhood typically present with more complicated and extensive disease course when compared to patients diagnosed in adulthood [7]. However, our knowledge of younger patients experiences with the disease is limited; there is a lack of published studies that have explored the lived experiences of children with CD or that have examined any differences between pediatric and adult CD [8–13]. Awareness of these differences and similarities is important, not only to ensure we have a comprehensive picture of the disease and its trajectory from childhood into later life, but to also ensure that the assessment of pediatric CD is relevant and meaningful. This is especially important in clinical trial settings where the ability of new drugs to improve the patient’s disease experience is under scrutiny.

For CD, the adult patient’s perspective on their disease is widely published [14]. The most common symptoms and burdens have been well-documented and the impact of CD on patients’ HRQL has been the focus of many studies [15–18]. However, as noted above, there is very little in the public domain which talks to the potentially unique experiences of children living with CD. A targeted review of the literature was conducted to identify relevant published studies. Five papers were found that reported qualitative data related to pediatric CD, however only one focused solely on CD [11]; two included children with CD or ulcerative colitis (UC) [8, 12] and two included children with CD or juvenile idiopathic arthritis [9, 10]. In Lynch & Spence’s (2008) qualitative study, four participants were interviewed about their experiences, however, although described as “adolescents”, these were older participants ranging from 16 to 21 years. Thus, further qualitative research was needed to address the gap in the literature and capture the range of symptoms and HRQL impacts associated with pediatric CD including those experienced by the very youngest children. This study was conducted to gain a better understanding of the lived experiences of children with CD.

## Methods

### Study design and participants

Recommendations in the field of pediatric outcomes research emphasize the importance of collecting information within narrow age bands, for example 2–4 years, 5–7 years, 8–11 years and 12–17 years (adolescents) [19]. Due to developmental differences associated with these childhood stages, it is important for researchers to examine potential differences in actual disease experiences as well as the reliability of children’s reporting between ages [19]. Thus, qualitative concept elicitation (CE) interviews were conducted with patients aged 5–7, 8–11 and 12–17 years and parents/caregivers (hereafter referred to as “parents”^1^) of children aged 2–4, 5–7 and 8–11 years. Some parents and children from the same family/household were recruited to allow for the conduct of dyad analysis.

Participants were identified using a purposive sampling approach via referrals from US-based clinicians in primary care or gastroenterology practices. Clinicians or site staff approached the parents of children with CD to ascertain their/their child’s interest in the study, provide them with information about the study and if permission was obtained, confirm eligibility of the child. Child participants were eligible to take part in the interview if they were 5–17 years old, fluent in US-English and had a diagnosis of CD confirmed by sigmoidoscopy or colonoscopy which had been active (of any severity) within the past 12 months. Child participants were excluded if they had a previous diagnosis of UC, indeterminate colitis, radiation colitis or diverticular-associated colitis, significant surgical resection, or uncontrolled psychiatric or physical comorbid condition. All child participants provided assent, either verbally (if 5–10 years old, which was also confirmed by the interviewer) or in writing (if 11–17 years old). If the parent was also recruited to participate, they completed a separate consent form. Parent participants were eligible if their child was 2–11 years old and met the same clinical criteria described above. Upon consent, the clinician or site staff completed a medical and health information form for the child with CD; demographic data were collected from either the participant (if > 12 years old) or the participant’s parent.

All study procedures were in accordance with ethical standards of the 1964 Helsinki Declaration and its later amendments, relevant laws, and institutional guidelines. The study protocol was approved by an independent institutional review board and participants received a stipend.

### Ethics approval

This study was conducted in compliance with Good Clinical Practice guidelines, including International Conference on Harmonization Guidelines [20]. In addition, all applicable local laws and regulatory requirements were adhered to throughout the study. Before recruiting participants, all study documents were submitted and approved by the Copernicus Group Independent Review Board®; approval number is 20182658.

### Interviews

All interviews were conducted one-to-one between interviewer and participant. Children aged 5–7 and 8–11 years were interviewed in-person, with their parent present but not actively participating in the interview. For children aged 12–17 years and parent participants, they were able to choose whether to be interviewed in-person or via telephone. In-person interviews took place in a conference room of a local hotel. Children 12–17 years old could also choose to have their parent present (albeit not actively participating) during the interview. Interviews were conducted by highly experienced interviewers who have substantial experience of qualitative interviewing. The interviews lasted no more than 45 min and were conducted using a semi-structured interview guide.

Prior to the interview day, child participants were asked to make a collage which represented their CD and have it available during the interview. The collage was used to build rapport with the child during the interview; children were asked to talk about the relevance and meaning of images and text included in their collage. Children were also asked to think of an animal that best represents their CD: “*If you think about your <Crohn’s> as being an animal, what would it be? Why is that?*”. This was a spontaneous question which participants were not prepared for; its purpose was to help children identify thoughts, feelings, and emotions and become reflective about their illness. The specific animal was not critical, rather, the concepts/experiences the selected animal represented. These creative tasks were not completed by parent participants.

In all interviews, questions were open-ended and broad allowing participants to discuss their/their child’s experiences in an unbiased, spontaneous way. If a participant mentioned a concept of relevance (i.e., symptom or HRQL impact related to CD), follow-up probes were used to explore the participant’s perception of the experience in greater depth. For the parent interviews, the interviewer focused discussion around observed behaviors and signs that indicate the presence of symptoms and impacts associated with CD to the parent. Children were able to use Play-Doh to model different stool consistencies if this was appropriate during the interview discussion.

### Sample size

The logic of qualitative sampling rests not on the number of participants interviewed but on the basis of ‘saturation’, that is, the point at which no new insights are likely to be obtained [21]. Therefore, sample size is not so much a criterion for judging the rigor of a sampling strategy, but, rather, for judging the extent to which issues of saturation have been explicitly thought through. Although we can never see the complete picture in qualitative work, we will know when we have reached a point of ‘saturation’ when nothing new emerges [22]. Therefore, based on published guidance [21] and the authors’ experience of how many interviews are typically needed to reach theoretical saturation in a relatively homogeneous population [22], it was anticipated that saturation would be met when approximately 30 children (aged 5–17 years old) and 20 parents had been interviewed.

### Analysis

All interviews were transcribed verbatim and analyzed using inductive thematic methods [23]. The coding of interview transcripts was conducted by two experienced qualitative researchers, who have undertaken many qualitative research projects and have years of expertise in qualitative coding analysis. Responses to the ‘animal’ question were subject to thematic analysis along with all other qualitative data analysis. All coding was facilitated by the qualitative software NVivo version 12. All data were de-identified before analysis was conducted. There were six steps to thematic analysis that were followed in the current study; although they may appear linear, this is a flexible and reflective process, which, if necessary, during the coding and analysis process steps, could be revisited [23]. The steps followed were:

1. Familiarization – reading and re-reading the transcripts and identifying meaningful segments of text;

2. Generating codes – coding distinct and relevant concepts systematically;

3. Identifying themes – collating codes into potential themes;

4. Reviewing themes – checking the themes work in relation to the coding, data, and research objectives;

5. Defining themes – refining the themes to make them specific and clear;

6. Report production – selecting clear and vivid examples that relate back to the research questions in a scholarly report.

Analysis of qualitative data was stratified into four age groups: 2–4 years, 5–7 years, 8–11 years and 12–17 years. These age groups reflect significant shifts in children’s cognitive development [19, 24] and are important to consider in the context of determining the ability to accurately self-report. Thus, the purpose of this stratification was to allow for comparison of themes reported between the four age groups (“age subgroup analysis”) as well as between parents versus children (“reporter type subgroup analysis”). A dyad analysis was conducted to further examine the reliability of reporting by age; whereas the two subgroup analyses focus on the consistency with which concepts were reported. In the dyad analysis, we examined the level of agreement in the reporting on the *presence* and *absence* of a concept by children and parents from the same household. This was important for the 5–7 and 8–11 age groups because it was less clear whether these children would be able to reliably report on their own disease experiences.

The accuracy of thematic analysis was confirmed by comparing independent themes identified by different researchers on a selection of transcripts. Coders regularly reviewed each other’s coding and discussed how codes were developed and applied consistently throughout the transcripts. Completed coding was given a final review for consistency and appropriateness.

Thematic saturation was analyzed for children and parents separately. To determine if saturation was met in this study, participants were divided into three equal sets based on the chronological order of when the participant was interviewed. Saturation of symptom concepts was analyzed for the child sample first and then compared to the parent sample. Saturation of HRQL impact concepts was then performed. Saturation was considered to have been met when no new concepts were discussed in the last set of interviews within each sample [21, 25]. If saturation was not met, interviews would continue until such time.

## Results

### Participant characteristics

In total, 31 children and 18 parent participants were recruited to take part in the interviews; no child or parent participant withdrew or dropped out of the study. Of the 18 parent participants, 11 had a child also participating in the study. Thus, there were 11 parent-child dyads interviewed. Participant-reported demographic data, broken down by age group, are presented in Table 1. Data relating to the children of the seven non-dyad parents were also collected; hence data is summarized for *N* = 38 children. Among the child sample, 19 (50%) were female and 25 (66%) were white. Among the parent sample, 15 (83%) were female and 11 (61%) were white. Most participants rated their/their child’s health as “good” or better (84%) and most rated their/their child’s CD as “mild” (42%) or “moderate” (50%).

**Table 1:** Demographic Characteristics of Children and Parent Participants^a^

	2–4 years		5–7 years		8–11 years		12–17 years		Total Child Sample	Total Parent Sample
	Child	Parent	Child	Parent	Child	Parent	Child	Parent		
**N**	**3**	**3**	**6**	**6**	**9**	**9**	**20**	**–**	**38**^**b**^	**18**
**Age**								**n/a**		
Mean (SD)	3.3 (0.58)	40.7 (1.53)	5.5 (0.84)	37.2 (4.36)	9.7 (1.12)	40.8 (6.18)	14.4 (1.39)		11.0 (4.19)	39.6 (4.02)
Median	3.0	41.0	5.0	36.5	9.0	40.0	14.0		12.0	39.2
Min - Max	3–4	39–42	5–7	32–45	8–11	31–49	12–17		3–17	31–49
Missing n	0	0	0	0	0	1	0		0	1
**Gender (n %)**								**n/a**		
Female	2 (66.67)	2 (66.67)	3 (50.00)	6 (100.00)	5 (55.56)	7 (77.78)	9 (45.00)		19 (50.00)	15 (83.33)
Male	1 (33.33)	1 (33.33)	3 (50.00)	0 (0.00)	4 (44.44)	2 (22.22)	11 (55.00)		19 (50.00)	3 (16.67)
**Ethnicity (n %)**								**n/a**		
Hispanic/Latino	0 (0.00)	0 (0.00)	1 (16.67)	1 (16.67)	0 (0.00)	0 (0.00)	2 (10.00)		3 (7.89)	1 (5.56)
Not Hispanic/Latino	3 (100.00)	3 (100.00)	5 (83.33)	5 (83.33)	9 (100.00)	9 (100.00)	18 (90.00)		35 (92.11)	17 (94.44)
**Race**								**n/a**		
White/Caucasian	2 (66.67)	2 (66.67)	2 (33.33)	2 (33.33)	7 (77.78)	7 (77.78)	14 (70.00)		25 (65.79)	11 (61.11)
Black/African American	1 (33.33)	1 (33.33)	3 (50.00)	3 (50.00)	1 (11.11)	1 (11.11)	2 (10.00)		7 (18.42)	5 (27.78)
American Indian/ Alaska Native	0 (0.00)	0 (0.00)	0 (0.00)	0 (0.00)	0 (0.00)	0 (0.00)	1 (5.00)		1 (2.63)	0 (0.00)
Asian	0 (0.00)	0 (0.00)	0 (0.00)	0 (0.00)	0 (0.00)	0 (0.00)	1 (5.00)		1 (2.63)	0 (0.00)
Other^c^	0 (0.00)	0 (0.00)	1 (16.67)	1 (16.67)	1 (11.11)	1 (11.11)	2 (10.00)		4 (10.53)	2 (11.11)
**Child’s current health (self/parent report)**		**n/a**		**n/a**		**n/a**		**n/a**		**n/a**
Excellent	0 (0.00)		1 (16.67)		0 (0.00)		3 (15.00)		4 (10.53)	
Very Good	0 (0.00)		0 (0.00)		2 (22.22)		2 (10.00)		4 (10.53)	
Good	3 (100.00)		3 (50.00)		6 (66.67)		12 (60.00)		24 (63.16)	
Fair	0 (0.00)		2 (33.33)		1 (11.11)		3 (15.00)		6 (15.79)	
Poor	0 (0.00)		0 (0.00)		0 (0.00)		0 (0.00)		0 (0.00)	
**Child’s current CD severity (self/parent report)**		**n/a**		**n/a**		**n/a**		**n/a**		**n/a**
Remission	0 (0.00)		0 (0.00)		0 (0.00)		1 (5.00)		1 (2.63)	
Mild	2 (66.67)		2 (33.33)		5 (55.56)		7 (35.00)		16 (42.11)	
Moderate	1 (33.33)		3 (50.00)		4 (44.44)		11 (55.00)		19 (50.00)	
Severe	0 (0.00)		1 (16.67)		0 (0.00)		1 (5.00)		2 (5.26)	
Very severe	0 (0.00)		0 (0.00)		0 (0.00)		0 (0.00)		0 (0.00)	
**CD flare in past 2 weeks**										
Yes	0 (0.00)		3 (50.00)		3 (33.33)		9 (45.00)		15 (39.47)	
No	3 (100.00)		3 (50.00)		6 (66.67)		11 (55.00)		23 (60.53)	
**Severity of most recent flare**		**n/a**		**n/a**		**n/a**		**n/a**		**n/a**
Very mild	0 (0.00)		0 (0.00)		0 (0.00)		2 (10.00)		2 (5.26)	
Mild	2 (66.67)		1 (16.67)		1 (11.11)		2 (10.00)		6 (15.79)	
Moderate	1 (33.33)		2 (33.33)		7 (77.78)		10 (50.00)		20 (52.63)	
Severe	0 (0.00)		1 (16.67)		1 (11.11)		2 (10.00)		4 (10.53)	
Very severe	0 (0.00)		1 (16.67)		0 (0.00)		1 (5.00)		2 (5.26)	
Missing	0 (0.00)		1 (16.67)		0 (0.00)		3 (15.00)		4 (10.53)	
**Highest level of education**	**n/a**		**n/a**		**n/a**		**n/a**	**n/a**	**n/a**	
Did not complete high school		0 (0.00)		0 (0.00)		0 (0.00)				0 (0.00)
High School Diploma/GED		0 (0.00)		1 (16.67)		2 (22.22)				3 (16.67)
4-Year College Degree		3 (100.00)		2 (33.33)		3 (33.33)				8 (44.44)
Graduate Degree or Higher		0 (0.00)		2 (33.33)		4 (44.44)				6 (33.33)
Other		0 (0.00)		1 (16.67)		0 (0.00)				1 (5.56)
**Employment status**	**n/a**		**n/a**		**n/a**		**n/a**	**n/a**	**n/a**	
Employed Full-Time		1 (33.33)		4 (66.67)		7 (77.78)				12 (66.67)
Employed Part-Time		0 (0.00)		0 (0.00)		2 (22.22)				2 (11.11)
Homemaker		1 (33.33)		2 (33.33)		0 (0.00)				3 (16.67)
Student		0 (0.00)		0 (0.00)		0 (0.00)				0 (0.00)
Retired		0 (0.00)		0 (0.00)		0 (0.00)				0 (0.00)
Unemployed		0 (0.00)		0 (0.00)		0 (0.00)				0 (0.00)
Other		0 (0.00)		0 (0.00)		0 (0.00)				0 (0.00)
Missing		1 (33.33)		0 (0.00)		0 (0.00)				1 (5.56)

^a^ Data for children < 12 years old were provided by parents

^b^ Data on the children (*n* = 7) of parent participants were collected but their children did not participate in the interviews. ^c^Other: *n* = 1 patient participant identified as half White/Caucasian and Black/African American; *n* = 1 patient participant report Hispanic; *n* = 1 patient participant identified as White/Caucasian and Indian; *n* = 1 patient participant did not wish to answer; *n* = 1 parent participant identified as half White/Caucasian and Black/African American; *n* = 1 parent participant reported Hispanic

Clinician-reported health and medical data for the children with CD are presented in Table 2. In the total child sample, the average number of months since diagnosis was 30.8 (range = 2–168), with the number of months increasing with each increasing age group. Clinician-reported severity of CD rated over the past 30 days was consistent with participants’ own ratings, with clinicians reporting that most children had “mild” (42%) or “moderate” (42%) CD. This was also consistent across the age groups. Similarly, most children (84%) had not been hospitalized in the past year, and only one child had been hospitalized more than once. The most common current treatment being taken by children was biologics (58%), while others managed their CD using vitamins/probiotics (37%) and through dietary modification (32%).

**Table 2:** Clinical Characteristics of Children with CD

N	2–4 years	5–7 years	8–11 years	12–17 years	Total Child Sample
	3	6	9	20	38
**Time since diagnosis (months)**					
Mean (SD)	9.3 (1.53)	11.0 (13.73)	12.9 (9.71)	48.0 (44.17)	30.8 (37.24)
Median	9.0	6.0	11.0	47.0	13.0
Min - Max	8–11	2–38	2–30	2–168	2–168
Missing n	0	0	0	0	0
**CGI- CD severity (Last 30 days) – Clinician-reported**					
Remission	0 (0.00)	0 (0.00)	1 (11.11)	3 (15.00)	4 (10.53)
Mild	3 (100.00)	4 (66.67)	4 (44.44)	5 (25.00)	16 (42.11)
Moderate	0 (0.00)	1 (16.67)	3 (33.33)	12 (60.00)	16 (42.11)
Severe	0 (0.00)	0 (0.00)	1 (11.11)	0 (0.00)	1 (2.63)
Very Severe	0 (0.00)	1 (16.67)	0 (0.00)	0 (0.00)	1 (2.63)
**Hospitalizations in past 12 months**					
1	0	1	1	3	5
2	0	1	0	0	1
0	3	4	8	17	32
**Hemoglobin levels**					
Mean (SD)	13.9 (0.35)	11.8 (1.03)	12.6 (1.52)	13.0 (0.99)	12.8 (1.19)
Median	13.9	11.4	13.0	13.1	13.0
Min - Max	14–14	11–13	10–14	11–15	10–15
Missing n	1	1	0	1	3
**Surgery for CD?**					
Yes	0 (0.00)	1 (16.67)	1 (11.11)	7 (35.00)^e^	9 (23.68)
No	3 (100.00)	5 (83.33)	8 (88.89)	13 (65.00)	29 (76.32)
**Surgery/Procedure type**^**e**^					
Fecal Resection	0 (0.00)	0 (0.00)	0 (0.00)	1 (5.00)	1 (2.63)
Endoscopy	0 (0.00)	1 (16.67)	1 (11.11)	5 (25.00)	7 (18.42)
**Comorbidities**					
Anemia	0 (0.00)	3 (50.00)	4 (44.44)	6 (30.00)	13 (34.21)
Nutritional disorders	0 (0.00)	1 (16.67)	1 (11.11)	2 (10.00)	4 (10.53)
Psychiatric disorder	0 (0.00)	1 (16.67)	2 (22.22)	1 (5.00)	4 (10.53)
Anal fissure/abscesses	0 (0.00)	1 (16.67)	0 (0.00)	3 (15.00)	4 (10.53)
Cardiac disease	0 (0.00)	0 (0.00)	1 (11.11)	0 (0.00)	1 (2.63)
Other	0 (0.00)	2 (33.33)	3 (33.33)	4 (20.00)	9 (23.68)
**Child Treatments**					
**Current Treatment**					
Biologics / Anti-TNF	0	5	6	11	22
Vitamins and Probiotics	3	3	2	6	14
Diet	3	1	2	6	12
Aminosalicylate	1	0	3	7	11
Immunomodulator	0	0	3	6	9
Proton-pump inhibitor	0	1	3	3	7
Corticosteroid Glucocorticoids	0	1	1	3	5
OTC Pain Killer	0	2	1	2	5
Stool softener or Laxative	0	2	1	0	3
Gastrointestinal Agent	0	0	0	3	3
Antibiotics	1	1	0	0	2
Antiemetic	0	0	1	1	2
Cognitive Behavioral Therapy	0	0	1	0	1
Other	0	0	0	1	1
Enteral Nutritional Therapy	0	0	0	0	0
**Previous Treatment**					
Biologics / Anti-TNF	0	0	1	2	3
Vitamins and Probiotics	0	0	1	1	2
Diet	0	0	1	2	3
Aminosalicylate	0	2	1	6	9
Immunomodulator	0	1	1	2	4
Proton-pump inhibitor	0	0	1	0	1
Corticosteroid Glucocorticoids	0	2	4	9	15
OTC Pain Killer	0	1	0	0	1
Stool softener or Laxative	0	1	2	0	3
Gastrointestinal Agent	0	0	0	0	0
Antibiotics	0	0	1	0	1
Antiemetic	0	1	1	0	2
Cognitive Behavioral Therapy	0	0	0	0	0
Other	0	0	0	0	0
Enteral Nutritional Therapy	0	1	0	0	1

^e^Surgery data are missing for a 15-year-old participant

### A patient-centered conceptual model of pediatric CD

**Fig. 1:**
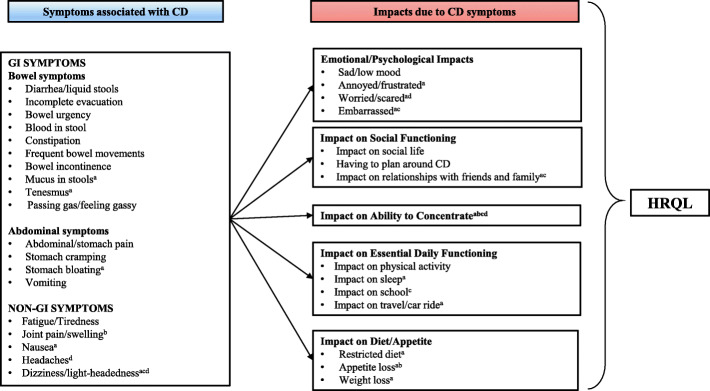
Patient-Centered Conceptual Model of Pediatric CD. Abbreviations: CD = Crohn’s disease; GI = gastrointestinal; HRQL = health-related quality of life. ^a^Not discussed by children 5–7 years old; ^b^not discussed by children 8–11 years old; ^c^not discussed by parents of children 2–4 years old; ^d^not discussed by parents of children 5–7 years old. Essential Daily Functioning refers to activities, functions, or practices necessary within a child/adolescent’s daily life

In the qualitative interviews, participants described their/their child’s experience of CD and it was clear that these children live with a disease that is both unpredictable and burdensome. Thematic analysis identified a range of symptoms, with those most frequently reported being abdominal or stomach pain, passing gas/feeling gassy, diarrhea or liquid stools, fatigue/tiredness, bowel urgency, blood in stools, stomach cramping, constipation, and incomplete evacuation. Both children and parents reported these symptoms at similar rates. As a result of these symptoms, and CD in general, participants described the negative consequences on their lives. Children and parents talked about being affected in terms of their/their child’s emotional and psychological well-being, social functioning, not being able to participate in daily activities, as well as other impacts. The burdensome nature of CD was also apparent in participants’ responses to the ‘animal’ question. Taken together, these findings were used to develop a conceptual model of pediatric CD (Fig. 1). The model provides a global picture of the pediatric experience of CD rather than indicating causation and relationships. Nineteen symptoms and 16 HRQL impacts were reported by at least 10% of the total study population (i.e., children and parents) and are presented in the conceptual model. Some concepts were unique to specific age groups and these are identified in the footnotes of the model.

### Findings relating to pediatric CD symptoms

The model shows the 19 symptoms categorized into two domains: GI symptoms and non-GI symptoms. In the GI symptoms domain, two sub-domains emerged, namely abdominal symptoms and bowel symptoms.

#### GI symptoms: abdominal symptoms

Four abdominal symptoms were identified in the qualitative analysis including abdominal/stomach pain, stomach cramping, stomach bloating and vomiting (Table 3). Abdominal/stomach pain was the only symptom reported by all 49 participants. It was described as occurring frequently – at least weekly for two-thirds of participants – and at a high intensity – described as “severe” by almost half. Many described this symptom using terms such as “*stomach hurts*” or is *“hurting*” (*N* = 23) or “*stomach pain*” (*N* = 5). Abdominal/stomach pain was portrayed as a stabbing, sharp, poking or jabbing sensation by 12 participants, while others said they/their child experiences a dull (*N* = 4), punching (*N* = 3) or cramping (N = 3) sensation. As well as hearing their child complain about abdominal/stomach pain, parents also talked about seeing certain behaviors which indicate their child is in pain. Twelve parents reported seeing their child put their hands on their stomach, while others reported seeing their child bent over or in a fetal position (*N* = 11). Some parents commented that when their child is having abdominal/stomach pain, they notice they will avoid physical tasks or eating (*N* = 9, respectively).

**Table 3:** Symptoms Reported by > 10% of Study Participants

	Children				Parents				Total (***N*** = 49)	Total %
	Age of child			Total (***N*** = 31)	Age of child			Total (***N*** = 18)		
	5–7 (***N*** = 3)	8–11 (***N*** = 8)	12–17 (***N*** = 20)		2–4 (***N*** = 3)	5–7 (***N*** = 6)	8–11 (***N*** = 9)			
Abdominal/stomach pain	3	8	20	31 (100%)	3	6	9	18 (100%)	49	100%
Passing gas/feeling gassy	3	8	17	28 (90%)	3	5	9	17 (94%)	45	92%
Diarrhea/liquid stools	3	6	18	27 (87%)	3	6	8	17 (94%)	44	90%
Fatigue/Tiredness	1	7	16	24 (77%)	2	6	9	17 (94%)	41	84%
Bowel urgency	3	6	19	28 (90%)	2	4	7	13 (72%)	41	84%
Blood in stools	2	5	18	25 (81%)	2	6	7	15 (83%)	40	82%
Stomach cramping	2	5	18	25 (81%)	3	4	8	15 (83%)	40	82%
Constipation	1	6	13	20 (65%)	3	6	9	18 (100%)	38	78%
Incomplete evacuation	2	8	12	22 (71%)	3	4	7	14 (78%)	36	73%
Frequent bowel movements	1	6	13	20 (65%)	1	4	5	10 (56%)	30	61%
Nausea	0	4	13	17 (55%)	3	4	5	12 (67%)	29	59%
Vomiting	2	3	9	14 (45%)	3	3	4	10 (56%)	24	49%
Stomach bloating	0	5	9	14 (45%)	2	4	4	10 (56%)	24	49%
Bowel incontinence	1	3	7	11 (35%)	1	6	2	9 (50%)	20	41%
Mucus in stools	0	2	8	10 (32%)	1	3	3	7 (39%)	17	35%
Joint pain/swelling	1	0	4	5 (16%)	1	3	2	6 (33%)	11	22%
Headaches	1	2	4	7 (23%)	1	0	3	4 (22%)	11	22%
Tenesmus	0	1	6	7 (23%)	0	1	1	2 (11%)	9	18%
Dizzy/light-headedness	0	2	3	5 (16%)	0	0	1	1 (6%)	6	12%

Stomach cramping (*N* = 40) and abdominal/stomach pain are conceptualized separately in the conceptual model (Fig. 1). While some participants described these symptoms as the same or very similar, for example some defined cramping as “*less severe stomach pain*”, others considered them distinct. As this 11-year old girl explained: *“I think when I hear abdominal pain, I think of like sharp pains …And then when I hear cramping, I feel like that’s more of a, like a dull almost like continuous movement.”*

Stomach bloating was reported by almost half of participants (*N* = 24). Participants described their stomach feeling “*big”, “tight”,” hard”, “full”* and *“swollen*” and indicated that the symptom is usually experienced after eating. It can be uncomfortable and even painful, as one 15-year old girl describes: “*It’s just, it’s really uncomfortable, like I can see it clearly… I can feel the extra air in my stomach.”* Interestingly, children aged 5–7 years did not report stomach bloating but four parents of children in this age group did identify it as a relevant.

#### 3.3.2. GI symptoms: bowel symptoms

There were 10 bowel symptoms reported by parents and children (Fig. 1), of which diarrhea or liquid stools was discussed most frequently (*N* = 44). It was described using terms such as “*diarrhea*” (*N* = 19), “*runny*” (N = 4), “*liquid*” (*N* = 3) “*watery*” (N = 3), and “*mushy*” (N = 3). Diarrhea or liquid stools was associated with children having stomach pain (*N* = 13), bloody stools (*N* = 10), stomach cramping (*N* = 8) and frequent bowel movements (N = 8). Parents reported knowing their child has diarrhea/liquid stools because they see the consistency of their child’s stool, or they see them rush to the bathroom or because of the timing of when their child goes to the bathroom, i.e., immediately after a meal. As well as diarrhea, constipation was a common symptom of CD (*N* = 38), with participants describing that they/their child may be “*blocked up*”, “*cannot go*”, “*compacted*” and “*poop not coming out*”. In addition to the child telling the parent, observable indicators of constipation included taking a long time in the bathroom (*N* = 4) or looking bloated (*N* = 2).

Bowel urgency was another important symptom (*N* = 41), described by children as “*not being able to hold it*”, having to “*run to the bathroom*”, feeling “*like you immediately need to go”* or needing to “*explode*”*.* By nature, it was described as an unpredictable symptom which “*comes out of nowhere*” and tends to occur more often when the child is having a flare, as this 14-year-old boy comments: “*It’s random. Sometimes it’s back-to-back. Sometimes they’re spread out. [It’s] kinda hard to plan my day”*. This unpredictability impacts children in myriad ways, for example this father of an 8-year-old girl noted: “*Many of times we’ve had to pull off on the side of the highway… and let her go because we know if we don’t, she’s gonna have an accident.”* The frequency of bowel urgency varied for children, although most described it as happening either daily (*N* = 10) or weekly (*N* = 11). In addition to having their child tell them about bowel urgency, for example, “*saying she has to poop and that it’s an emergency*”, eight parents commented that they see their child “*rush to the bathroom*”.

Parents and children also reported seeing blood in their/their child’s stool (*N* = 40) or on occasion seeing blood on the toilet paper (*N* = 5) or in the toilet bowl itself (N = 5). Four parents mentioned that their child will bring them to the bathroom to look at the toilet paper or bowl if this happens. For some participants, this was a scary or worrying symptom of CD, as this 11-year-old boy explains: *“I flipped out and went to the nurse at school”*. Having to make frequent trips to the bathroom to poop was reported by *N* = 30 participants, and this was described as being worse when they/their child was having a flare (*N* = 12) or was experiencing diarrhea (*N* = 8) or stomach pain (N = 8). Passing gas/feeling gassy was also an important symptom (*N* = 45) although it was described as less severe compared with other GI symptoms. In contrast to stomach bloating, which participants described feeling in the stomach area, passing gas/feeling gassy was experienced primarily in the lower GI and intestinal area. Indeed, the symptoms overlap as participants commented that having gas could be related to abdominal symptoms including stomach pain (*N* = 7) and bloating (*N* = 5).

#### Non-GI symptoms

Of the non-GI symptoms captured in the conceptual model (Fig. 1), fatigue/tiredness was the most significantly mentioned (*N* = 41). As well as talking about “*tired or tiredness*”, participants also mentioned having “*less energy*” (*N* = 3), “*wanting to lay down or sleep*” (N = 3), “*feeling fatigued*” (*N* = 2), “*weak*” (N = 3), and “*feeling exhausted*” (*N* = 1). This 14-year-old boy comments on how he is affected by fatigue: “*It’s kinda hard for me to go anywhere. I usually have to run off to the bathroom a lot. And I’m always just really tired*”. This was a persistent symptom, with 10 participants reporting that it happens daily and a further ten describing it as “*severe*”. The substantial impact of Crohn’s fatigue is well-captured by this 14-year-old girl who states: *“I do get tired a lot… Doesn’t matter how much I sleep, I’m, like, tired…I’d wake up and I feel like I need to go back to bed…. I’m always tired, even if it’s just a little bit”.*

### Findings relating to pediatric HRQL impacts due to CD

HRQL impacts due to CD are conceptualized in the conceptual model into five domains which includes impacts relating to emotional or psychological well-being, social functioning, ability to concentrate, essential daily functioning, and diet/appetite. (Fig. 1; Table 4). Overall, the most commonly discussed impacts were related to essential daily functioning, defined as ‘activities, functions or practices that are necessary within a child’s or adolescent’s daily life’. Within this domain, impact on physical activity was the most significantly discussed (*N* = 33). Participants described difficulty participating a range of activities such as sports, exercise, gym class as well as walking. Walking was mentioned as both exercise and a function of general mobility, such as walking from class to class. Some participants reported that they/their child avoid physical activities because of CD as explained by this 13-year-old boy: *“It makes me not want to do things, like if I even, if I wanted to play uh, soccer with my friend, I have to go to the bathroom quick, and… I feel that I don’t want to do it because I just, uh, in the bathroom for too long…”* These limitations were described in relation to CD as a whole, as well as, in relation to specific symptoms including stomach pain (*N* = 12), tiredness (*N* = 6) and joint pain (*n* = 5). Interestingly, children aged 5–7 years did not report any physical impact but two parents of children in this age group did identify it as a relevant.

**Table 4:** HRQL Impacts Reported by ≥10% of Study Participants

	Children				Parents				Total (***N*** = 49)	Total %
	Age of child			Total (***N*** = 31)	Age of child			Total (***N*** = 18)		
	5–7 (***N*** = 3)	8–11 (***N*** = 8)	12–17 (***N*** = 20)		2–4 (***N*** = 3)	5–7 (***N*** = 6)	8–11 (***N*** = 9)			
Impact on physical activity	0	6	18	24 (77%)	1	2	6	9 (50%)	33	67%
Impact on social life	1	5	11	17 (55%)	3	4	5	12 (67%)	29	59%
Impact on school	2	6	10	18 (58%)	0	3	8	11 (61%)	29	59%
Sad/low mood	2	4	10	16 (52%)	3	2	4	9 (50%)	25	51%
Annoyed/frustrated	0	3	12	15 (48%)	2	2	2	6 (33%)	21	43%
Restricted diet	0	3	10	13 (42%)	1	2	5	8 (44%)	21	43%
Worried/scared	0	2	9	11 (35%)	2	0	2	4 (22%)	15	31%
Appetite loss	0	0	7	7 (23%)	1	2	5	8 (44%)	15	31%
Impact on sleep	0	2	5	7 (23%)	1	3	3	7 (39%)	14	29%
Having to plan around CD	0	1	5	6 (19%)	1	2	2	5 (28%)	11	22%
Relationships with friends and family	0	1	4	5 (16%)	0	1	3	4 (22%)	9	18%
Weight loss	0	1	2	3 (10%)	1	1	4	6 (33%)	9	18%
Embarrassed	0	2	3	5 (16%)	0	1	2	3 (17%)	8	16%
Impact on traveling/car ride	0	1	2	3 (10%)	1	1	2	4 (22%)	7	14%
Impact on ability to concentrate	0	0	3	3 (10%)	0	0	2	2 (11%)	5	10%

The impact on children’s school life was another clear theme in the interviews (*N* = 29). Participants noted that CD has led them/their child to missing school due to CD and to a lesser extent, affected their/their child’s actual performance at school. This appeared to be a particular concern for parents as it was their second most mentioned impact (*N* = 11). Children miss school to attend doctor’s visits, hospital appointments or because they are suffering with symptoms such as stomach pain and tiredness. In terms of cognitive function, five participants also mentioned having an impact on their ability to concentrate which was categorized as a stand-alone domain. They described difficulty focusing during general day-to-day activities, and also in the context of doing homework and being able to follow the lesson when in class.

Social functioning was an important area of concern for children and parents. While there was discussion about its impact on relationships as well as having to plan around CD, the main concern related to their/their child’s social life (*N* = 29). Participants described this in terms of having difficulty engaging with friends or participating in “fun” activities. Some children and parents commented that these problems resulted from a lack of motivation or willingness to participate, which is summarized by this 14-year old girl *“It’s like I just kind of don’t wanna do anything when I’m feeling it [stomach pain]. I just wanna like, lay down and wait for it to go away”.* However, others talked about how the impact of CD on their/their child’s social life was more physical than mental. The main symptoms that children and parents attributed these issues to were stomach pain (*N* = 10) and tiredness (*N* = 5).

Diet and appetite were mentioned by both parents and children (*N* = 21). Participants explained how their/their child’s diet had changed to avoid triggering symptoms (e.g., diarrhea, bowel urgency) when outside the home or exacerbating current symptoms. For example, this mother of a 9-year old girl stated: *“she didn’t want to eat because she said once she ate, she would have to go to the bathroom…So she stopped wanting to eat because she didn’t want to go to the bathroom”*. CD appears to impact both the range of food and volume of food that children can eat. Some participants discussed loss of appetite in general (*N* = 8) or food avoidance due to symptoms like stomach pain, nausea, mouth sores, or being in a flare (*N* = 7), and as a result, the impact it has on children’s weight (*N* = 9).

The data demonstrated that this disease has a serious impact on children’s HRQL. Over half of all participants (*N* = 25) reported they/their child experiences sadness and low mood due to having CD including participants from all age groups. Five participants talked about their/their child crying, with one 12-year-old boy feeling like he is missing out on life: “*When I’m feeling down, I’m thinking to myself that I’m the only one that has this problem…And I don’t know anybody else that has this problem”*. Children and parents also discussed how CD makes them/their child feel anxious, annoyed, frustrated, and embarrassed. Children and parents said they/their child found CD in general “*annoying*” or “*frustrating*”, while others mentioned feeling “*mad*”, “*irritated*”, “*bothered*”, “*angry*” and “*cranky*” about specific symptoms including stomach pain (*N* = 8) and CD flares (*N* = 5).

### Subgroup findings

#### Age cohorts

Qualitative analysis of concepts identified in the interviews based on the four age groups revealed that children’s experience of the symptoms and HRQL impacts associated with CD were largely consistent across the age groups. In general, GI symptoms were the most consistent by age, whereas symptoms conceptualized in the ‘non-GI’ domain showed slightly more age variability (Table 3). With respect to children aged 2–4-years old, for whom only their parents were interviewed, all symptoms included in the conceptual model were mentioned except for dizziness/light-headedness and tenesmus. More importantly, no unique symptoms or impacts were identified for this age group. Similarly, in the 5–7 age group, neither children nor parents reported dizziness/light-headedness. A further four symptoms failed to be mentioned by these children (including stomach bloating, mucus in stools, tenesmus, nausea) but they were reported by the parents of 5–7-year-olds. With respect to children aged 8–11-years old, all symptoms were mentioned by the children and parents except for joint pain/swelling which was not reported by any child aged 8–11. All symptoms were reported by children in the adolescent age group.

Analysis also revealed a good level consistency of HRQL impacts by age (Table 4). As before, all impacts were reported by children in the adolescent age group and all impacts were reported for children aged 8–11, except for two which children specifically did not report (ability to concentrate and appetite loss) albeit that parents of 8–11-year-olds did report these. There was slightly more variability in the younger age groups. For example, 12 of the 15 impact concepts were not reported by a single 5–7-year-old child, despite the majority of these being mentioned by parents of 5–7-year-olds. In addition, four concepts were not identified as relevant for children aged 2–4 (including embarrassed, relationships with friends and family, school life, ability to concentrate).

#### Reporter type

Qualitative analysis of the concepts identified in the interviews based on reporter type revealed that, in general, parents and children have a consistent understanding and experience of the symptoms and impacts associated with CD. Interestingly, parent participants talked about their knowledge of their child’s symptoms and impacts being largely based on the child informing them but in some cases, it was also based on observing specific behaviors or signs which indicate the presence of a symptom or impact. Analysis revealed no symptom or HRQL impact concept was identified by parent participants alone, i.e., all parent-reported concepts were identified by at least one child participant. There was one symptom, headaches, which was reported by children aged 5 to 7-years old which was not mentioned by any parents of children this age, but it was mentioned by children, and parents of children, of other ages.

### Dyad findings

The level of agreement on concept presence/absence within each dyad was examined to consider the reliability of reporting among children aged 5–11 years. Eleven child-parent dyads were included in the study including three dyads in the 5–7 age group and eight dyads in the 8–11 age group. Overall, there was a high level of reporting agreement across all dyads. Of the 19 symptoms, there was 100% dyad agreement for two symptoms (abdominal/stomach pain and passing gas/feeling gassy) and > 70% agreement for a further nine symptoms. Bowel urgency, frequent bowel movements and stomach bloating were the only symptoms with < 50% dyad agreement. While none of the 15 HRQL impacts were associated with 100% dyad agreement, > 50% of dyads agreed on the presence/absence of 11 impact concepts. Physical activity, feeling annoyed/frustrated, having a restricted diet and feeling sad/low mood had < 50% dyad agreement. Interestingly, dyad agreement was higher among the 8–11 dyads compared with the 5–7 dyads for symptom reporting (average 77% vs. 61%, respectively), but lower for impact reporting (average 56% vs. 67%, respectively). A dyad analysis results table is provided in Supplementary Table 1.

### Saturation findings

In the child sample, saturation analysis indicated that no new symptom concept was reported for the first time in the last set of child interviews. In the parent sample, dizziness/light-headedness was identified for the first time by one parent who was in the last set of parent interviews indicating this concept did not meet the threshold for saturation. However, dizziness/light-headedness was described in detail by five children, therefore further exploration was not deemed necessary and concept saturation of symptom concepts was considered met across the total study population. Regarding HRQL impact concepts, no new concept was mentioned for the first time in the last set of child or parent interviews, thus concept saturation of impact concepts was achieved. Saturation analysis results tables are provided in Supplementary Tables 2 and 3.

## Discussion

This research has revealed the substantial burden of CD on children’s lives. This is one of the first studies of pediatric CD which has comprehensively explored, through in-depth qualitative interviews, the symptomatic and quality of life experiences among children from as young as age 2, up to 17 years of age. In addition, the inclusion of parents of children with CD in the study offered a unique perspective supplementary to the patient perspective; it not only provided additional context and richness to the data but also helped to examine the validity of reporting from children themselves. The conceptual model of pediatric CD shows the range and relative importance of the core symptoms and HRQL impacts associated with pediatric CD, which represents an important step towards improving our overall understanding of this illness, especially with respect to the very youngest children.

The interview findings demonstrated that the symptoms and HRQL impacts associated with pediatric CD were, in general, consistent across the age spectrum based on both child and parent reporting. Although not all concepts were reported by parents and children to the same extent, the same symptoms were included in the “top eight” by both reporter groups, albeit the ordering of frequency was slightly different (Table 3). A similar pattern was found for HRQL impact concepts (Table 4). With respect to two oldest age groups, children’s descriptions revealed that the experiences of adolescents and those aged 8–11 years were highly similar. Adolescents reported all concepts in the conceptual model. Children aged 8–11 reported all but one symptom (joint pain/swelling) and all but two impacts (appetite loss, ability to concentrate). However, parents of children aged 8–11 described all concepts. One symptom, dizziness/light-headedness, appeared to be more strongly associated with CD in children in the older two age groups (i.e., 8–17 years) since it was not reported by any child, or parent of a child, under 8 years old. Dizziness is not a well-documented symptom of CD but could be related to medication side-effects or dehydration from CD-related diarrhea [26].

The experiences of children aged 2–4-years old were based on parent-reporting alone and were also highly consistent with those reported by/for older children. Although two symptoms (dizziness/light-headedness and tenesmus) and four HRQL impacts (feeling embarrassed, impact on relationships, ability to concentrate and school) were not described by parents of children in this age group, when considering the nature of these experiences, their absence appears rationale. For instance, children of this age are unlikely to be attending school and embarrassment is a high-level emotion and not commonly observed in children of this age. Furthermore, there is considerable variability regarding a child’s ability to concentrate and focus attention within the first 4 years of life [27]. Lastly, children aged 2–4 years tend to have more momentary playmates than traditional “friendships” [28] and it is not until they are older that they start forming more long-term, meaningful relationships. In the 5–7 age group, the frequency of concepts reported was a little more variable. In general, we found children and parents in this age group reported fewer symptoms and HRQL impacts than participants in the other age groups and there was a larger discrepancy between parents and children aged 5–7. For instance, while five symptoms (mucus in stools, tenesmus, stomach bloating, nausea, dizziness/light-headedness) were not mentioned by any child aged 5–7, parents of children in this age group identified four of these as relevant. These may have been described by the 5–7-year-old children as part of other symptoms (for instance, bloating described as stomach pain, gas or cramping) or they may be too complex for children of this age to discuss (e.g. tenesmus, mucus in stools). Similarly, 12 of the 15 impact concepts were absent in the descriptions from these children but again, ten of these were reported by parents of children aged 5–7 years. These findings suggest that children aged 5–7 years and parents of children this age have slightly differing perspectives, or that children this age are not yet able to clearly or fully articulate their experiences of CD.

In terms of examining the reliability of reporting of children across the age spectrum, the interview data demonstrated that children in the oldest age group (12–17 years) were able to describe their CD-related experiences with relative ease. In addition, parents of children in the youngest age group (2–4 years) were able to provide detailed and rich descriptions of their child’s CD. Importantly, these parents did not mention any symptom or HRQL impact which was not already reported by participants in the older age groups indicating equivalence in experience between the youngest children and their older counterparts. From a measurement perspective, these findings support the suitability of self-reporting among children aged 12–17 and observer-reporting among parents of children aged 2–4. In the age groups where we interviewed parents and children (i.e. 5–7 and 8–11), qualitative analysis revealed equivalence in experience among children aged 8–11 compared with children in the other age groups, and between experience reported by the children and by the parents of children aged 8–11. On this basis, it would appear that either self-reporting or observer-reporting would be suitable from a measurement perspective. In the 5–7-year old age group, observer-reporting appeared to be more appropriate compared to patient self-reporting. There was greater discrepancy between parent and child reporting; parents reported more comprehensively, and in general, reading and language skills are less -developed in children of this age [17]. The dyad analysis also showed that, in general, agreement between patient and parent on symptom reporting was higher in the 8–11 age group vs. the 5–7 age group but lower for impact reporting. This may reflect the notion that as children get older, they gain more independence from their parents and the child’s quality of life becomes more difficult for the parent to know. However, based on the small sample size for this age group, no significant conclusions about these differences can be made.

These findings present a novel conceptualization of pediatric CD which appears to have been overlooked in the scientific literature. We have addressed that gap by focusing on a pediatric population which includes children as young as two and avoids confounding by collecting data only from those with CD (and not similar conditions such as UC). This has also provided opportunity to explore qualitative experiences within narrow age groups to consider the reliability of reporting from children across the age spectrum that will ultimately help to inform future measurement strategies. The findings are also of value to clinical practice allowing clinicians to better understand the language children and their parents use to describe their experiences which may aid in clinician-patient interactions as well as clinical decision-making.

## Limitations

It is important to acknowledge some study limitations. Firstly, the number of participants interviewed reflected a typical sample size for qualitative research of this nature [29], particularly in a rare condition. However, only three 5–7-year-old children were recruited; thus, the sample size in this age group was relatively small compared to the other age groups. As a result, we relied more heavily on insight from parents of children in this age group which may have led to the finding that there was greater discrepancy between reporter types which ultimately led to the recommendation to use observer-reporting for children of this age. Secondly, the majority of the patient population were considered to have mild to moderate CD, with only two children in the severe or very severe category. We believe that the difficulty faced trying to identify pediatric patients with severe disease reflects the fact that in general practice children diagnosed with CD are treated quickly and proactively to reduce symptoms and worsening of the disease [30]. Lastly, it requires mentioning that participants were recruited from the US only and interviews were conducted in US-English only, so consideration should be taken in assuming that the results are generalizable to other countries, languages or cultures.

We recognize that in light of these study limitations, future research is needed that attempts to capture the voice of these harder-to-reach patients. The results of this study lay the foundation for new measurement approaches including the development of a Crohn’s-specific outcome measure that could be used in future pediatric CD studies.

## Conclusions

These qualitative interviews revealed the substantial burden of pediatric CD from the patient and parent-observer perspective. The resultant conceptual model reflects the lived experience of this condition as reported by children and their parents. In a clinical trial setting, assessment of pediatric CD should consider the ability and ease with which children can describe and report on their disease experiences and choice of measurement should ensure that data reliability and accuracy is optimal.

## Supplementary Information

**Additional file 1. **Crohn’s Disease Concept Elicitation Supplement. **Supplementary Table 1A.** Analysis of Dyad Agreement for Symptom Concepts. **Supplementary Table 1B.** Analysis of Dyad Agreement for HRQL Impact Concepts. **Supplementary Table 2A.** Saturation of Symptom Concepts for Child Sample. **Supplementary Table 2B**. Saturation of Symptom Concepts for Parent Sample. **Supplementary Table 3A.** Saturation of HRQL Impact Concepts for Child Sample. **Supplementary Table 3B**. Saturation of HRQL Impact Concepts for Parent Sample.

## Data Availability

The datasets generated and/or analyzed during the current study are not publicly available due to the sensitive nature of the questions asked in this study but are available from the corresponding author on reasonable request.

## Abbreviations

CD: Crohn’s disease
CE: Concept elicitation
GI: Gastrointestinal
HRQL: Health-related quality of life
UC: Ulcerative colitis

## Footnotes

1 All parents/caregivers participating in the study were parents
