# Supplementary material for: A qualitative study to explore symptoms and impacts of pediatric and adolescent Crohn’s disease from patient and caregiver perspective
Source: J Patient Rep Outcomes. 2021 Jun 25;5:49. doi: 10.1186/s41687-021-00321-1 (PMC8233440; doi:10.1186/s41687-021-00321-1)
Supplement: Supplementary file 1 — Additional file 1. Crohn’s Disease Concept Elicitation Supplement. Supplementary Table 1A. Analysis of Dyad Agreement for Symptom Concepts. Supplementary Table 1B. Analysis of Dyad Agreement for HRQL Impact Concepts. Supplementary Table 2A. Saturation of Symptom Concepts for Child Sample. Supplementary Table 2B. Saturation of Symptom Concepts for Parent Sample. Supplementary Table 3A. Saturation of HRQL Impact Concepts for Child Sample. Supplementary Table 3B. Saturation of HRQL Impact Concepts for Parent Sample. [file 41687_2021_321_MOESM1_ESM.docx]

**Crohn’s Disease Concept Elicitation Supplement**

**Supplementary Table 1A.** **Analysis of Dyad Agreement for Symptom Concepts**

|  | **Percentage Agreement Within Each Dyad** | | **Total % Agreement**  **(N = 11)** |
| --- | --- | --- | --- |
|  | **5–7 years**  **(N = 3)** | **8–11 years**  **(N = 8)** |  |
| Abdominal/stomach pain | 100% | 100% | 100% |
| Passing gas/feeling gassy | 100% | 100% | 100% |
| Diarrhea/liquid stools | 100% | 88% | 91% |
| Joint pain/swelling | 100% | 88% | 91% |
| Dizzy/light-headedness | 100% | 88% | 91% |
| Incomplete evacuation | 67% | 88% | 82% |
| Vomiting | 33% | 100% | 82% |
| Fatigue/Tiredness | 33% | 88% | 73% |
| Blood in stools | 67% | 75% | 73% |
| Stomach cramping | 67% | 75% | 73% |
| Tenesmus | 67% | 75% | 73% |
| Constipation | 33% | 75% | 64% |
| Nausea | 33% | 75% | 64% |
| Bowel incontinence | 33% | 75% | 64% |
| Mucus in stools | 67% | 63% | 64% |
| Headaches | 67% | 63% | 64% |
| Bowel urgency | 33% | 50% | 45% |
| Frequent bowel movements | 33% | 50% | 45% |
| Stomach bloating | 33% | 50% | 45% |

**Supplementary Table 1B.** **Analysis of Dyad Agreement for HRQL Impact Concepts**

|  | **Percentage Agreement Within Each Dyad** | | **Total % Agreement**  **(N = 11)** |
| --- | --- | --- | --- |
|  | **5–7 years**  **(N = 3)** | **8–11 years**  **(N = 8)** |  |
| Worried/scared | 100% | 75% | 82% |
| Embarrassed | 100% | 75% | 82% |
| Impact on school | 100% | 75% | 82% |
| Impact on ability to concentrate | 100% | 75% | 82% |
| Having to plan around CD | 67% | 63% | 64% |
| Impact on traveling/car ride | 67% | 63% | 64% |
| Impact on social life | 67% | 50% | 55% |
| Relationships with friends and family | 67% | 50% | 55% |
| Appetite loss | 67% | 50% | 55% |
| Impact on sleep | 33% | 63% | 55% |
| Impact on physical activity | 67% | 38% | 45% |
| Annoyed/frustrated | 33% | 38% | 36% |
| Restricted diet | 33% | 38% | 36% |
| Sad/low mood | 33% | 25% | 27% |

**Supplementary Table 2A.** **Saturation of Symptom Concepts for Child Sample**

| **Concept** | **Set 1**  **(N = 10)** | **Set 2**  **(N = 10)** | **Set 3**  **(N = 11)** | **Saturation Met** |
| --- | --- | --- | --- | --- |
| Abdominal/stomach pain | ✓ | ✓ | ✓ | ✓ |
| Passing gas/feeling gassy | ✓ | ✓ | ✓ | ✓ |
| Diarrhea/liquid stools | ✓ | ✓ | ✓ | ✓ |
| Incomplete evacuation | ✓ | ✓ | ✓ | ✓ |
| Fatigue/Tiredness | ✓ | ✓ | ✓ | ✓ |
| Bowel urgency | ✓ | ✓ | ✓ | ✓ |
| Blood in stools | ✓ | ✓ | ✓ | ✓ |
| Stomach cramping | ✓ | ✓ | ✓ | ✓ |
| Constipation | ✓ | ✓ | ✓ | ✓ |
| Tenesmus | ✓ | X | ✓ | ✓ |
| Frequent bowel movements | ✓ | ✓ | ✓ | ✓ |
| Nausea | ✓ | ✓ | ✓ | ✓ |
| Vomiting | ✓ | ✓ | ✓ | ✓ |
| Stomach bloating | ✓ | ✓ | ✓ | ✓ |
| Bowel incontinence | ✓ | ✓ | ✓ | ✓ |
| Mucus in stools | ✓ | ✓ | ✓ | ✓ |
| Headaches | ✓ | ✓ | ✓ | ✓ |
| Joint pain/swelling | ✓ | ✓ | ✓ | ✓ |
| Dizzy/light-headedness | X | ✓ | ✓ | ✓ |

**Supplementary Table 2B.** **Saturation of Symptom Concepts for Parent Sample**

| **Concept** | **Set 1**  **(N = 6)** | **Set 2**  **(N = 6)** | **Set 3**  **(N = 6)** | **Saturation Met** |
| --- | --- | --- | --- | --- |
| Abdominal/stomach pain | ✓ | ✓ | ✓ | ✓ |
| Passing gas/feeling gassy | ✓ | ✓ | ✓ | ✓ |
| Diarrhea/liquid stools | ✓ | ✓ | ✓ | ✓ |
| Incomplete evacuation | ✓ | ✓ | ✓ | ✓ |
| Fatigue/Tiredness | ✓ | ✓ | ✓ | ✓ |
| Bowel urgency | ✓ | ✓ | ✓ | ✓ |
| Blood in stools | ✓ | ✓ | ✓ | ✓ |
| Stomach cramping | ✓ | ✓ | ✓ | ✓ |
| Constipation | ✓ | ✓ | ✓ | ✓ |
| Frequent bowel movements | ✓ | ✓ | ✓ | ✓ |
| Nausea | ✓ | ✓ | ✓ | ✓ |
| Vomiting | ✓ | ✓ | ✓ | ✓ |
| Stomach bloating | ✓ | ✓ | ✓ | ✓ |
| Bowel incontinence | ✓ | ✓ | ✓ | ✓ |
| Mucus in stools | ✓ | ✓ | ✓ | ✓ |
| Joint pain/swelling | ✓ | ✓ | ✓ | ✓ |
| Headaches | X | ✓ | ✓ | ✓ |
| Tenesmus | ✓ | ✓ | X | ✓ |
| Dizzy/light-headedness | X | X | ✓ | X |

**Supplementary Table 3A. Saturation of HRQL Impact Concepts for Child Sample**

| **Concept** | **Set 1**  **(N = 10)** | **Set 2**  **(N = 10)** | **Set 3**  **(N = 11)** | **Saturation Met** |
| --- | --- | --- | --- | --- |
| Impact on physical activity | ✓ | ✓ | ✓ | ✓ |
| Impact on school | ✓ | ✓ | ✓ | ✓ |
| Impact on social life | ✓ | ✓ | ✓ | ✓ |
| Sad/low mood | ✓ | ✓ | ✓ | ✓ |
| Annoyed/frustrated | ✓ | ✓ | ✓ | ✓ |
| Restricted diet | ✓ | ✓ | ✓ | ✓ |
| Worried/scared | ✓ | ✓ | ✓ | ✓ |
| Appetite loss | ✓ | ✓ | ✓ | ✓ |
| Impact on sleep | ✓ | ✓ | ✓ | ✓ |
| Having to plan around CD | ✓ | ✓ | ✓ | ✓ |
| Embarrassed | X | ✓ | ✓ | ✓ |
| Impact on relationships with friends/family | ✓ | ✓ | ✓ | ✓ |
| Weight loss | ✓ | X | ✓ | ✓ |
| Impact on traveling/car ride | X | ✓ | ✓ | ✓ |
| Impact on ability to concentrate | ✓ | ✓ | X | ✓ |

**Supplementary Table 3B. Saturation of HRQL Impact Concepts for Parent Sample**

| **Concept** | **Set 1**  **(N = 6)** | **Set 2**  **(N = 6)** | **Set 3**  **(N = 6)** | **Saturation Met** |
| --- | --- | --- | --- | --- |
| Impact on social life | ✓ | ✓ | ✓ | ✓ |
| Impact on school | ✓ | ✓ | ✓ | ✓ |
| Sad/low mood | ✓ | ✓ | ✓ | ✓ |
| Impact on physical activity | ✓ | ✓ | ✓ | ✓ |
| Restricted diet | ✓ | ✓ | ✓ | ✓ |
| Appetite loss | ✓ | ✓ | ✓ | ✓ |
| Impact on sleep | ✓ | ✓ | ✓ | ✓ |
| Annoyed/frustrated | ✓ | ✓ | ✓ | ✓ |
| Weight loss | ✓ | ✓ | ✓ | ✓ |
| Having to plan around CD | ✓ | ✓ | ✓ | ✓ |
| Worried/scared | ✓ | ✓ | X | ✓ |
| Relationships with friends and family | ✓ | X | ✓ | ✓ |
| Impact on traveling/car ride | ✓ | ✓ | ✓ | ✓ |
| Embarrassed | X | ✓ | ✓ | ✓ |
| Impact on ability to concentrate | ✓ | X | ✓ | ✓ |
